# In Vitro Validation of Size-Dependent Antiviral Activity of *Phaeodactylum tricornutum*-Derived Peptide Fractions Against SARS-CoV-2

**DOI:** 10.3390/md24040122

**Published:** 2026-03-25

**Authors:** David Mauricio Cañedo-Figueroa, Blanca Azucena Márquez-Reyna, Alan Orlando Santos-Mena, Daniela Nahomi Calderón-Sandate, Flor Itzel Lira-Hernández, Julio E. Castañeda-Delgado, Ana Cristina García-Herrera, Rosa María del Ángel, Moisés León-Juárez, Marco Antonio Valdez-Flores, Gabriela López-Angulo, Claudia Desireé Norzagaray-Valenzuela, Loranda Calderón-Zamora, Evelin Cervantes-Bobadilla, Juan Fidel Osuna-Ramos, Luis Adrián De Jesús-González

**Affiliations:** 1 Laboratorio de Virología Molecular, Unidad de Investigación Biomédica de Zacatecas, Instituto Mexicano del Seguro Social, Zacatecas 98000, Mexico; david.canedo.fm@uas.edu.mx (D.M.C.-F.); blanca.marquezr@uap.uaz.edu.mx (B.A.M.-R.); alan.santos.mena@outlook.com (A.O.S.-M.); 20202095@uaz.edu.mx (D.N.C.-S.); flor.lihe@gmail.com (F.I.L.-H.); ana.garciaher@imss.gob.mx (A.C.G.-H.); 2 Laboratorio de Virología y Diseño de Antivirales, Facultad de Medicina, Universidad Autónoma de Sinaloa (UAS), Culiacan 80246, Mexico; 3 Investigadores por México (SECIHTI), Unidad de Investigación Biomédica de Zacatecas, Instituto Mexicano del Seguro Social, Zacatecas 98000, Mexico; julioenriquecastaneda@gmail.com; 4 Department of Infectomics and Molecular Pathogenesis, Center for Research and Advanced Studies (CINVESTAV-IPN), Mexico City 07360, Mexico; rmangel@cinvestav.mx; 5 Laboratorio de Virología Perinatal y Diseño Molecular de Antígenos y Biomarcadores, Departamento de Inmunobioquimica, Instituto Nacional de Perinatología “Isidro Espinosa de los Reyes”, Mexico City 11000, Mexico; moisesleoninper@gmail.com; 6 Faculty of Medicine, Autonomous University of Sinaloa, Culiacan 80246, Mexico; marco.valdez@uas.edu.mx (M.A.V.-F.); gabylopez@uas.edu.mx (G.L.-A.); claudia.norzagaray@uas.edu.mx (C.D.N.-V.); loranda.calderon@uas.edu.mx (L.C.-Z.); evelin.cervantes.fm@uas.edu.mx (E.C.-B.)

**Keywords:** *SARS-CoV-2*, antiviral peptides, *Phaeodactylum tricornutum*, marine microalgae, peptide fractions, in vitro antiviral activity, Omicron variant

## Abstract

The continuous emergence of SARS-CoV-2 variants highlights the need for novel antiviral agents with favorable safety profiles. Marine microalgae constitute a valuable source of bioactive compounds, including antiviral peptides. Building on previous *in silico* identification of peptides derived from the marine microalga *Phaeodactylum tricornutum* with predicted activity against SARS-CoV-2, this study evaluated the antiviral capacity of peptide fractions generated by enzymatic hydrolysis and separated by molecular weight (10–30, 5–10, 3–5, and <3 kDa) in human alveolar epithelial A549 cells infected with the SARS-CoV-2. Cytotoxicity analyses, assessed using MTT and resazurin assays, revealed a moderate, concentration-dependent reduction in metabolic activity while maintaining overall cell viability within an acceptable range for antiviral evaluation, with higher-molecular-weight fractions (10–30 and 5–10 kDa) displaying the most stable profiles. Antiviral activity was assessed by flow cytometry following post-infection treatment. Lower-molecular-weight fractions (3–5 and <3 kDa) showed early reductions in infection at low concentrations but exhibited variable responses. In contrast, the 10–30 and 5–10 kDa fractions showed more robust, dose-dependent inhibition at medium and high concentrations, reducing infection levels to levels close to those observed in uninfected controls. Comparative analysis with the reference antiviral drug lopinavir demonstrated that peptide fractions exhibit lower cytotoxicity while retaining antiviral activity under equivalent experimental conditions. Overall, these results indicate that antiviral efficacy is strongly influenced by peptide molecular weight and consistency of response. This work provides experimental in vitro validation of *P. tricornutum*–derived peptide fractions as marine antiviral candidates and supports the integration of in silico and functional approaches for marine drug discovery.

## 1. Introduction

Since the end of 2019, the emergence of Severe Acute Respiratory Syndrome Coronavirus 2 (SARS-CoV-2) has posed an unprecedented global health challenge. Following its identification in China, SARS-CoV-2 was recognized as the causative agent of Coronavirus Disease 2019 (COVID-19), leading the World Health Organization to declare a global pandemic in early 2020 [1,2]. To date, COVID-19 has resulted in more than 700 million confirmed cases and over 7 million deaths worldwide [3].

The rapid development and deployment of effective vaccines significantly reduced hospitalization and mortality rates associated with COVID-19 [4]. However, the continuous emergence of viral variants driven by SARS-CoV-2’s high mutability [5], together with recurrent outbreaks and the limited availability of early outpatient antiviral treatments capable of eliminating infection at early stages, underscores the persistent need for novel antiviral strategies [6,7,8].

In this context, natural products have gained renewed attention as sources of antiviral compounds due to their structural diversity, generally low cytotoxicity, reduced production costs, and compatibility with scalable biotechnological processes [9]. Among these, bioactive peptides have emerged as desirable therapeutic candidates. Advances in bioinformatics, rational design, sequence optimization, and computational filtering have enabled the identification of peptides with high specificity and efficacy across multiple disease settings [10,11].

Computational approaches have facilitated the in silico evaluation of peptide–target interactions, highlighting antiviral peptides (AVPs) as promising molecules that can interfere with key stages of viral infection [12,13]. Importantly, several of these computational predictions have been successfully translated into experimental models, reinforcing the validity of this discovery strategy. Examples include AVPs that inhibit viral fusion during measles virus infection [14], cyclic peptides targeting SARS-CoV-2 entry mechanisms [15], and amphibian- or fungal-derived peptides that exhibit antiviral and immunomodulatory activities [16,17].

Marine microalgae are a particularly promising yet underexplored source of antiviral peptides. Marine microalgae are a particularly promising yet underexplored source of antiviral peptides. Among them, *Phaeodactylum tricornutum* is a marine diatom belonging to the class of unicellular photosynthetic microorganisms, widely distributed in marine ecosystems. Diatoms are characterized by their silica-based cell walls and high metabolic versatility, which enables the production of diverse bioactive compounds, including proteins, lipids, and bioactive peptides of potential pharmacological interest. *P. tricornutum* has gained increasing attention due to its rapid growth, relatively simple cultivation, and high protein content, making it a suitable candidate for the discovery of biologically active peptides [18,19].

On the other hand, *P. tricornutum*, a diatom with well-established biotechnological relevance, has demonstrated in vitro antiviral activity against dengue virus serotype 2 [18]. More recently, our group reported an in silico screening of peptides derived from *P. tricornutum*, identifying candidate sequences with favorable binding affinity, molecular stability, and interactions with catalytic residues of the SARS-CoV-2 main protease (Mpro) [19].

Building directly upon these computational findings, the present study represents the in vitro experimental validation of *P. tricornutum*-derived peptide fractions obtained by molecular weight separation. Specifically, protein lysates were fractionated into four peptide groups (10–30 kDa, 5–10 kDa, 3–5 kDa, and <3 kDa), which were subsequently evaluated for cytotoxicity and antiviral activity against SARS-CoV-2 Omicron variant JN.1 in human alveolar epithelial A549 cells. By assessing size-dependent antiviral effects, this work bridges in silico predictions with functional in vitro validation and provides experimental evidence supporting *P. tricornutum* as a source of antiviral peptide fractions.

## 2. Results

### 2.1. Evaluation of the Cytotoxicity of Peptide Fractions of Phaeodactylum tricornutum

In previous studies conducted by our group, we demonstrated that protein and peptide fractions derived from *P. tricornutum* exhibit low cytotoxicity and a favorable safety profile in in vitro models against the dengue virus. In these models, the higher molecular weight fractions showed minimal impact on the viability of Huh-7 hepatocellular carcinoma cells, even at high concentrations [18]. Furthermore, subsequent in silico analyses identified peptides derived from this microalga with structural and energetic properties favorable for interaction with relevant SARS-CoV-2 viral targets, suggesting a peptide-size-dependent antiviral potential [19].

Based on this background, the present study was designed as an in vitro experimental validation focused on SARS-CoV-2, beginning with the systematic evaluation of the cytotoxicity profiles of peptide fractions separated by molecular weight, as an essential preliminary step prior to the analysis of their antiviral activity.

**Figure 1:**
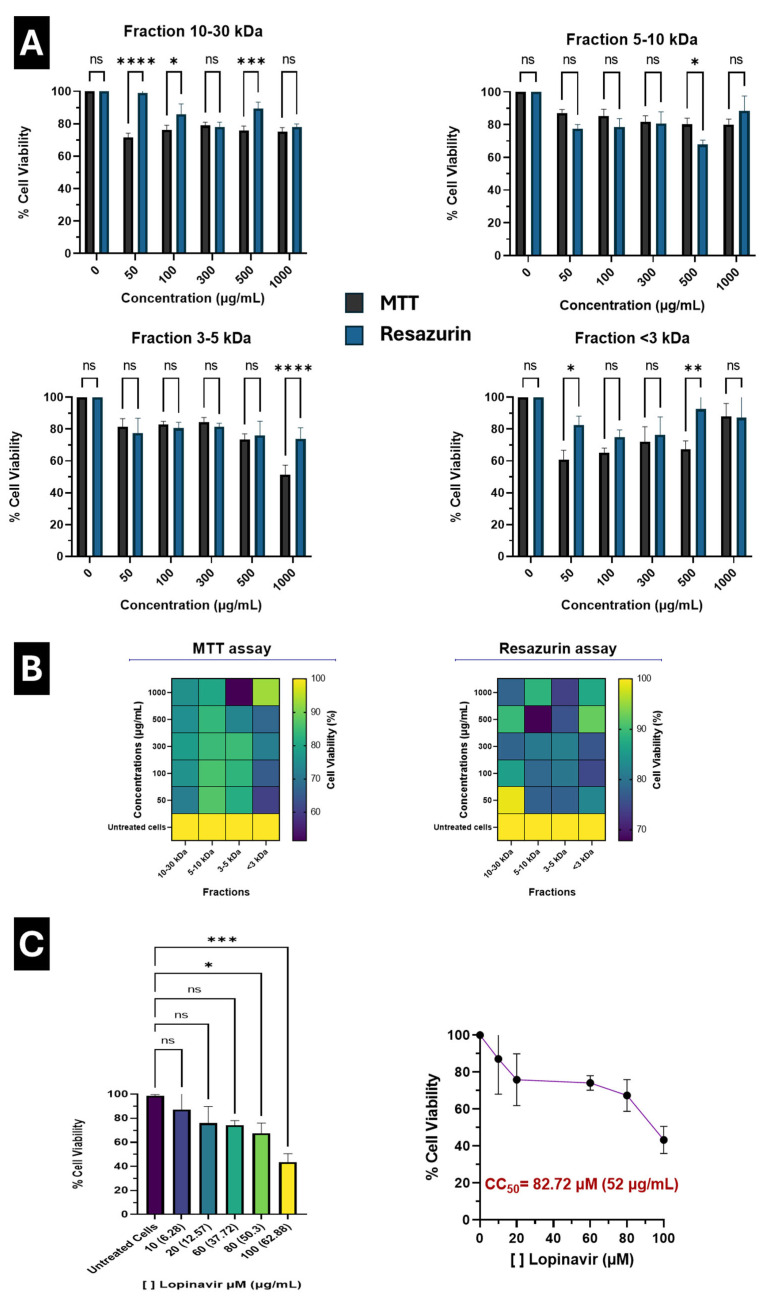
Cytotoxicity assessment of *P. tricornutum* peptide fractions in A549 cells. (**A**) Percentage of cell viability of human alveolar epithelial A549 cells treated with peptide fractions of different molecular weight ranges (10–30 kDa, 5–10 kDa, 3–5 kDa, and <3 kDa) across a concentration range of 50–1000 µg/mL. Cell viability was evaluated using MTT and resazurin assays. Differences between MTT and resazurin assays at each concentration were evaluated using two-way ANOVA followed by Šídák’s multiple comparisons test. (**B**) Heatmap representation of cell viability percentages for all peptide fractions at the indicated concentrations. Cell viability was normalized to untreated cells. Data are presented as mean ± standard deviation. (**C**) Cytotoxicity of lopinavir used as a reference control, evaluated at different concentrations expressed in µM and µg/mL. Differences among concentrations were analyzed using one-way ANOVA followed by Tukey’s post hoc test. CC_50_ was determined by nonlinear regression analysis using a dose–response (variable slope) model. Statistical significance is indicated as follows: * *p* < 0.05, ** *p* < 0.01, *** *p* < 0.001, **** *p* < 0.0001; ns, not significant.

The cytotoxicity of the 10–30 kDa, 5–10 kDa, 3–5 kDa, and <3 kDa fractions was evaluated in human A549 alveolar epithelial cells across a broad concentration range (25–1000 µg/mL). As shown in **Figure 1A**, the 10–30 kDa fraction exhibited a stable cell viability profile across the tested concentrations, with values consistently remaining above approximately 70%. Similarly, the 5–10 kDa fraction showed low cytotoxicity, maintaining cell viability above 80% even at the highest concentrations evaluated.

To strengthen the cytotoxicity assessment, two complementary assays were performed: MTT and resazurin reduction (**Figure 1A**). The MTT assay evaluates mitochondrial metabolic activity by reducing tetrazolium salts, whereas the resazurin assay measures global cellular metabolic activity via redox reactions [20,21,22,23]. Both methods yielded comparable results across all peptide fractions and concentrations, with only minor differences (<10%) observed at specific concentrations. These variations did not affect the overall interpretation of low cytotoxicity, supporting the robustness of the findings.

In contrast, the 3–5 kDa fraction displayed concentration-dependent reductions in cell viability at higher doses, reaching approximately 51–73.9% (MTT and resazurin assays, respectively) at 1000 µg/mL, indicative of moderate cytotoxicity at elevated concentrations. Notably, the <3 kDa fraction exhibited distinct behavior: greater variability at lower concentrations, followed by an increase in cell viability as the concentration increased, with higher values observed at the upper end of the tested range. These patterns are summarized in **Figure 1B**, where a clear size-dependent trend is observed, with the higher-molecular-weight fractions (10–30 and 5–10 kDa) exhibiting the most favorable and consistent safety profiles.

Additionally, morphological evaluation of the cell monolayer was performed to assess potential structural alterations induced by peptide treatment. As shown in **Supplementary Figure S1**, no evident changes in cell morphology, detachment, or monolayer disruption were observed across any of the tested concentrations or fractions, supporting the absence of cytotoxic effects at the structural level.

As a reference compound, lopinavir, an antiviral drug previously reported to exhibit activity against SARS-CoV-2, was included as a control [24,25]. Lopinavir was evaluated in parallel using concentrations expressed in both µM and µg/mL to enable direct comparison with peptide fractions, which are reported in µg/mL (**Figure 1C**). In contrast to peptide fractions, lopinavir exhibited a clear concentration-dependent cytotoxic effect, allowing the determination of a CC_50_ value of 82.72 µM (52 µg/mL).

Importantly, none of the peptide fractions reached a 50% reduction in cell viability, even at the highest concentration tested (1000 µg/mL), indicating CC_50_ values greater than 1000 µg/mL for all fractions. This contrasts with the higher cytotoxicity observed for lopinavir and highlights the broader safety margin of the peptide fractions derived from *P. tricornutum*.

Because none of the evaluated fractions produced a consistent 50% reduction in cell viability across the analyzed concentration range, it was not possible to robustly calculate CC_50_ values. Overall, these results indicate that peptide fractions derived from *P. tricornutum* exhibit low intrinsic cytotoxicity, even at high concentrations. Taken together, these findings extend and confirm our group’s previous observations in other viral models and support the use of *P. tricornutum* peptide fractions, particularly those of higher molecular weightas safe candidates for further antiviral evaluation against SARS-CoV-2.

### 2.2. In Vitro Antiviral Capacity of Peptide Fractions from P. tricornutum Against SARS-CoV-2

As previously mentioned, our group’s earlier work demonstrated that protein fractions derived from *P. tricornutum* exhibit antiviral activity against dengue virus and display low cytotoxicity in various in vitro cell models. Furthermore, in silico approaches identified peptides derived from this microalga with high antiviral potential against SARS-CoV-2, characterized by high binding affinity and sustained structural stability against key viral targets, particularly the main protease (Mpro).

These computational analyses showed that the peptides A0A8J9SA87 and A0A8J9SDW0 formed stable interactions with essential catalytic residues of Mpro, including Cys145, His41, Glu166, and Phe140, which are determinants of Mpro’s enzymatic activity. Molecular dynamics simulations at 100 ns confirmed the high conformational stability of the complexes formed by these peptides, solidifying them as the most promising candidates within the *P. tricornutum* peptide repertoire. Taken together, these in silico results suggested that peptides of greater length and structural complexity could exhibit greater antiviral efficacy by promoting more stable and multivalent interactions with Mpro.

Based on the experimental and in silico background previously established by our group, the antiviral activity of peptide fractions from *P. tricornutum*, separated by molecular weight, was evaluated in vitro against SARS-CoV-2 infection. Antiviral activity was determined by flow cytometry, quantifying the proportion of cells positive for the Spike protein after infection with the Omicron JN.1 variant.

Representative flow cytometry dot plots are presented in **Supplementary Figure S2**, including mock-infected cells, infected untreated controls, infected cells treated with peptide fractions, and infected cells treated with lopinavir as a reference antiviral control. Lopinavir concentrations were selected based on cytotoxicity assays, using doses that maintained at least 80% cell viability (maximum concentration of 18 µM, equivalent to 11.32 µg/mL), ensuring that antiviral effects were not attributable to cytotoxicity.

At 100 µg/mL, the 3–5 kDa and <3 kDa fractions showed a marked reduction in the SARS-CoV-2-positive cell population compared to the infected control, while the 10–30 kDa and 5–10 kDa fractions exhibited a more moderate decrease in the signal associated with viral infection. However, when the concentration was increased to 300 and 500 µg/mL, the 10–30 kDa and 5–10 kDa fractions produced a substantial reduction in the infected cell population, with minimal residual signal. In contrast, the lower-molecular-weight fractions showed variable responses.

**Figure 2:**
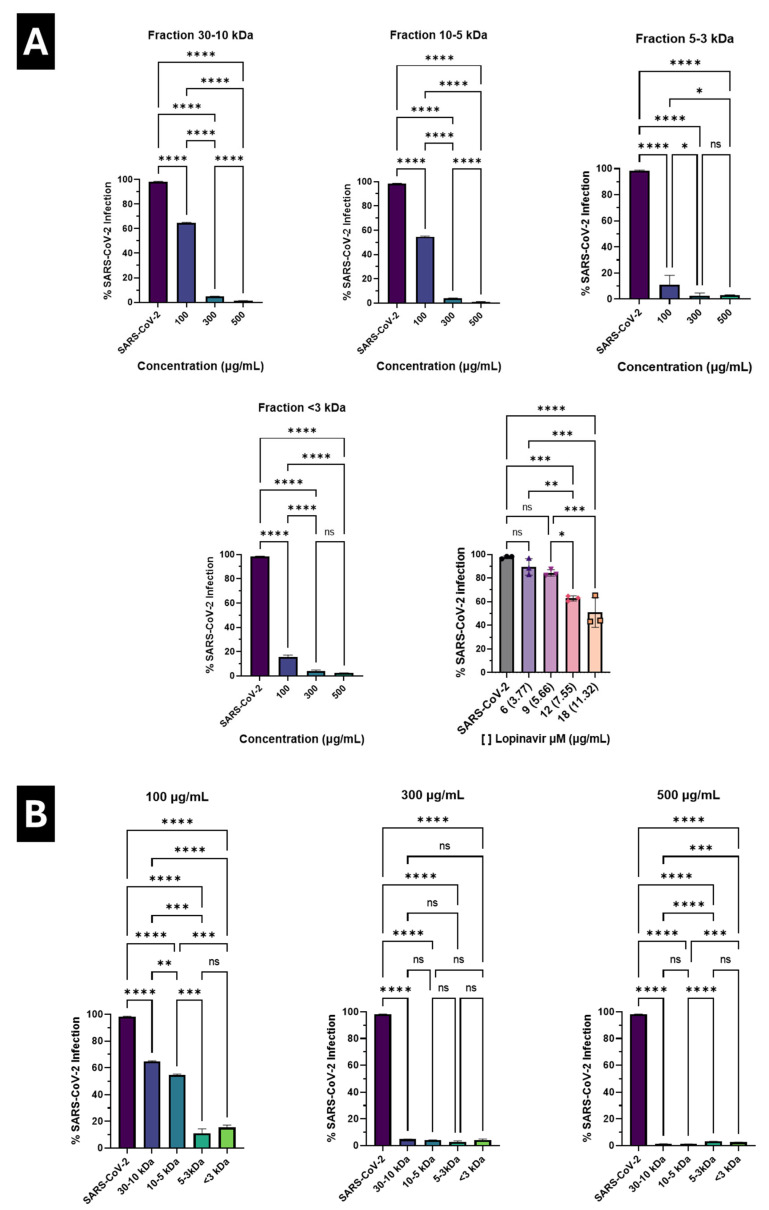
Antiviral activity of *P. tricornutum* peptide fractions and lopinavir against SARS-CoV-2 in A549 cells. (**A**) Percentage of SARS-CoV-2–infected cells after post-infection treatment with peptide fractions (10–30 kDa, 5–10 kDa, 3–5 kDa, and <3 kDa) at concentrations of 100, 300, and 500 µg/mL, and with lopinavir (control) at the indicated concentrations expressed in µM and µg/mL. (**B**) Comparison of infection percentages grouped by concentration across all peptide fractions. Data are expressed as mean ± standard deviation. Statistical significance was determined by one-way ANOVA followed by Tukey’s post hoc test. * *p* < 0.05, ** *p* < 0.01, *** *p* < 0.001, **** *p* < 0.0001; ns, not significant.

The post-treatment infection percentage for each fraction is shown in **Figure 2A**. At low concentrations, the 3–5 kDa and <3 kDa fractions showed a significant decrease in infection rate. However, this effect did not increase consistently with increasing concentration. In contrast, the 10–30 kDa fraction showed a progressive and dose-dependent reduction in the percentage of infected cells, reaching levels close to those observed in uninfected cells at 300 and 500 µg/mL. Similarly, the 5–10 kDa fraction showed a significant, dose-dependent antiviral effect, although of lesser magnitude than that of the higher-molecular-weight fraction.

On the other hand, **Figure 2B** shows the results grouped by concentration, allowing for direct comparison of the relative antiviral efficacy of the different fractions at equivalent doses. At 100 µg/mL, the 3–5 kDa and <3 kDa fractions showed a more pronounced reduction in the percentage of infection than the higher molecular weight fractions. However, at 300 and 500 µg/mL, the 10–30 kDa and 5–10 kDa fractions showed more robust and consistent inhibition of viral infection, clearly outperforming the lower-molecular-weight fractions.

To quantitatively compare antiviral activity and safety, CC_50_, IC_50_, and selectivity index (SI) values were estimated for each peptide fraction and for lopinavir as a reference control (**Table 1**). While CC_50_ and IC_50_ values for lopinavir could be accurately determined (CC_50_ = 82.72 µM [52 µg/mL], IC_50_ = 17.39 µM [10.9 µg/mL], SI = 4.77), peptide fractions did not reach clear 50% cytotoxicity or inhibition thresholds within the tested concentration range. Therefore, CC_50_ and IC_50_ values for peptide fractions are reported as approximate ranges or limits (e.g., >1000 µg/mL or <100 µg/mL). Notably, all peptide fractions maintained cell viability above 50% even at the highest concentrations tested, while still exhibiting antiviral activity, resulting in selectivity indices that are comparatively more favorable than those of the control.

Taken together, these results position *P. tricornutum* peptide fractions as promising antiviral candidates, with a more favorable balance between antiviral efficacy and cytotoxicity than the reference drug lopinavir under the evaluated conditions.

## 3. Discussion

Marine-derived biomaterials have become a strategic source of bioactive compounds with antiviral applications due to their high structural diversity, potential for sustainable production, and often favorable safety profiles. In this context, AVPs have emerged as an exceptionally versatile class, capable of interfering with different stages of the viral replication cycle, including entry, membrane fusion, and inhibition of essential viral enzymes [26,27]. In the case of SARS-CoV-2, various endogenous peptides and peptide derivatives, such as defensins and cathelicidins, have been shown to possess antiviral activity, mediated by mechanisms including modulation of the Spike-ACE2 interaction or interference with post-entry intracellular processes [28,29]. Furthermore, multiple studies have highlighted that the antiviral efficacy of peptides depends critically on their molecular architecture, such as length, net charge, amphipathicity, and structural complexity, and that this activity can be enhanced through chemical or structural modifications, such as multimerization or lipidation, as observed for defensin-derived peptides and fusion inhibitors like EK1 and its optimized variant EK1C4 [28,30,31,32,33]. This background provides a solid conceptual framework for exploring naturally occurring peptide fractions as complex reservoirs of antiviral activity, particularly when integrated with computational screening strategies.

In this study, peptide fractions derived from *P. tricornutum* were evaluated using a sequential experimental approach, beginning with the characterization of their cytotoxicity profile and subsequently with the functional validation of their in vitro antiviral activity against SARS-CoV-2 variant Omicron JN.1. Cytotoxicity analyses showed that the higher molecular weight fractions (10–30 and 5–10 kDa) exhibited the most stable and favorable safety profiles in A549 cells, maintaining high levels of cell viability across a wide concentration range. In contrast, the 3–5 kDa fraction showed a concentration-dependent decrease in cell viability at high doses, reaching approximately 51% at 1000 µg/mL, suggesting moderate cytotoxicity. Notably, the <3 kDa fraction showed non-monotonic behavior, with greater variability at low concentrations and a progressive increase in cell viability as concentration increased.

Importantly, differences between the cytotoxicity profiles observed in this study and those we previously reported by Rivera-Serrano et al. (2024) [18] may be explained using distinct cellular models. While Rivera-Serrano et al. evaluated peptide fractions in Huh-7 hepatocellular carcinoma cells, the present study employed A549 human alveolar epithelial cells. These cell lines differ substantially in their metabolic activity, mitochondrial dynamics, susceptibility to stress, and response to bioactive compounds, which may account for the observed differences in baseline viability and dose–response behavior. In particular, epithelial lung-derived cells such as A549 are known to exhibit distinct metabolic adaptations compared to hepatic-derived cell lines, potentially influencing the readout of metabolic-based viability assays such as MTT and resazurin.

Importantly, cell viability was assessed using both MTT and resazurin assays, which rely on different biochemical principles. While MTT measures mitochondrial metabolic activity by reducing tetrazolium salts to formazan crystals, resazurin is a redox indicator that reflects cellular metabolic capacity by converting to resorufin. The consistency observed between both assays, with differences generally below 10%, supports the robustness of the cytotoxicity data and reduces the likelihood of assay-dependent bias, as previously reported in comparative studies of viability assays [20,21,22,23].

This type of atypical dose–response has been previously described in toxicology and cell pharmacology studies. It has been associated with hormetic phenomena, adaptive mechanisms of cellular overcompensation, or effects dependent on the cell line and the complexity of the peptide mixture [34,35]. Although the present study did not address the mechanisms underlying this behavior, its observation is relevant, as it underscores the importance of peptide size and fraction composition when interpreting in vitro safety profiles.

Importantly, compared with the reference antiviral drug lopinavir [24,25], peptide fractions exhibited a markedly lower cytotoxicity profile. While lopinavir showed a defined CC_50_ value (82.72 µM; 52 µg/mL), peptide fractions did not reach 50% cytotoxicity within the evaluated concentration range (>1000 µg/mL), highlighting their broader safety margin under the same experimental conditions.

From an antiviral perspective, flow cytometry results revealed that the peptide fractions of *P. tricornutum* exhibit distinct inhibition profiles that depend on both molecular weight and concentration. At low concentrations (100 µg/mL), the lower-molecular-weight fractions (3–5 and <3 kDa) showed an early and marked reduction in the percentage of infected cells, suggesting that some low-molecular-weight components may exert a rapid antiviral effect at relatively low exposures. However, this effect did not increase consistently with increasing concentration and showed greater experimental variability. In contrast, the higher molecular weight fractions (10–30 and 5–10 kDa) showed a more robust and reproducible dose-dependent antiviral inhibition pattern, particularly at 300 and 500 µg/mL, reaching infection levels close to those observed in uninfected cells, especially in the case of the 10–30 kDa fraction. This behavior suggests that although smaller peptides can induce early inhibition, medium- to high-molecular-weight fractions provide more consistent and sustained antiviral inhibition as concentration increases, a finding particularly relevant from a translational perspective.

In this context, the inclusion of lopinavir as a reference control [24,25] allows a comparative interpretation of antiviral efficacy under standardized conditions. Although lopinavir displayed measurable antiviral activity within a defined concentration range that preserved ≥80% cell viability, peptide fractions achieved comparable or greater reductions in infection at higher concentrations while maintaining low cytotoxicity, suggesting a more favorable balance between efficacy and safety.

These findings gain greater significance when integrated with the experimental and computational background previously generated by our group [19]. Previous work demonstrated that the protein and peptide fractions of *P. tricornutum* exhibit antiviral activity against dengue virus and low cytotoxicity profiles in in vitro cell models [18]. Subsequently, using in silico approaches, peptides derived from this microalga were identified with high binding affinity and structural stability against the main protease of SARS-CoV-2 (Mpro), including stable interactions with essential catalytic residues such as Cys145, His41, Glu166, and Phe140, which were confirmed by molecular dynamics simulations. Although these computational analyses do not allow for the direct assignment of these peptides to a specific fraction based on molecular weight, the observation that larger fractions exhibit more consistent antiviral inhibition is conceptually congruent with the hypothesis that longer and more structurally complex peptides can establish more stable multivalent interactions with key viral targets, such as Mpro, thus favoring more robust functional inhibition.

Another relevant aspect to consider is the influence of the hydrolysis and fractionation process on the generation of the observed profiles. The literature has extensively demonstrated that the hydrolytic enzyme used, as well as the degree and duration of hydrolysis, significantly influence the length, hydrophobicity, and bioactivity of the resulting peptides, potentially generating very different functional profiles even from the same protein source [36,37]. In this sense, the variability observed between fractions could reflect differences in stability, susceptibility to degradation, interaction with cell membranes, or affinity for viral targets, especially in the lower-molecular-weight fractions [38].

From a pharmacological perspective, a key observation of this study is that CC_50_ and IC_50_ values for peptide fractions could not be precisely determined within the tested concentration range because no clear 50% cytotoxicity or inhibition thresholds were observed. Rather than representing a limitation, this reflects the low intrinsic cytotoxicity and broad therapeutic window of these fractions. In contrast, lopinavir exhibited well-defined CC_50_ and IC_50_ values, allowing the calculation of a selectivity index (SI = 4.77). When considering the estimated ranges for peptide fractions (CC_50_ > 1000 µg/mL and IC_50_ values within or below the tested range), their inferred selectivity indices suggest a more favorable safety–efficacy profile than that of the reference compound.

Based on the results obtained, future studies should focus on several complementary lines of inquiry. First, it will be essential to identify and characterize the individual peptides present in the most promising fractions using proteomic techniques (LC-MS/MS), followed by antiviral evaluation of the purified or synthesized peptides to confirm their specific contribution to the observed activity. Secondly, the implementation of biochemical assays targeting Mpro will enable experimental validation of in silico predictions and establish a direct functional relationship between enzyme inhibition and reduced cellular infection. Furthermore, additional time-course studies and mechanistic assays will help elucidate whether the antiviral activity is primarily associated with inhibition of viral entry, post-entry processes, or both. Finally, evaluation in additional cell models and, eventually, in three-dimensional systems or in vivo models, will be key to advancing the preclinical validation of these marine peptides as potential antiviral agents.

Collectively, these findings support the notion that peptide fractions derived from *P. tricornutum* not only retain antiviral activity but also exhibit a broader therapeutic window than a clinically used antiviral reference [39], reinforcing their potential as candidates for further development.

Taken together, this study reinforces the value of *P. tricornutum* as a relevant marine source of bioactive peptides with antiviral potential. It demonstrates that an integrated approach combining marine bioprospecting, in silico analysis, and in vitro functional validation constitutes a robust strategy for discovering new therapeutic candidates against SARS-CoV-2 and other emerging viruses.

## 4. Materials and Methods

### 4.1. Study Design and Compound Selection

This study was designed as an in vitro experimental validation of antiviral peptide-fraction candidates derived from *P. tricornutum*, following a previously published in silico screening pipeline targeting the SARS-CoV-2 main protease (Mpro) [19]. In the computational phase, peptides obtained from *P. tricornutum* were prioritized based on predicted binding affinity, interactions with key catalytic residues, and molecular stability. Here, the candidates were subsequently evaluated in vitro to validate their antiviral activity.

### 4.2. Preparation and Fractionation of P. tricornutum Peptides

Dried biomass of *P. tricornutum* (175 g) was subjected to desalting and defatting using ethyl acetate. Proteins were extracted by alkaline solubilization using NaOH solution (0.1 M, pH 10.0). The biomass was suspended in the alkaline solution at a 1:10 (*w*/*v*) ratio and incubated under constant agitation at room temperature for 1 h to facilitate protein solubilization. Insoluble material was removed by centrifugation at 10,000× *g* for 15 min, and the supernatant containing soluble proteins was collected. Protein precipitation was subsequently performed by isoelectric precipitation through pH adjustment using HCl.

Total soluble protein concentration was determined using a bicinchoninic acid (BCA) assay (Pierce™ BCA Protein Assay Kit, Thermo Fisher Scientific, Waltham, MA, USA; Cat. No. A55865), according to the manufacturer’s instructions.

Protein hydrolysis was performed using Alcalase^®^ enzyme (Alcalase^®^ 2.4 L FG, Novozymes A/S, Bagsværd, Denmark) at 4% (*w*/*w*) relative to total protein content for 1 h at 50 °C under alkaline conditions (pH 9.0). Following enzymatic hydrolysis, the reaction was terminated by heat inactivation at 95 °C for 10 min under acidic conditions (pH ≈ 4.5), ensuring complete protease denaturation and stabilization of the generated peptide mixture.

The resulting hydrolysates were fractionated by tangential flow ultrafiltration using molecular-weight-cutoff membranes (Amicon ^®^ Ultra-15 (Millipore, Billerica, MA, USA)) to obtain four peptide fractions: 10–30 kDa, 5–10 kDa, 3–5 kDa, and <3 kDa. This ultrafiltration step contributes to the removal of low-molecular-weight components, including residual salts and buffer constituents derived from the hydrolysis process. Fractions were lyophilized and resuspended in sterile distilled water to a final concentration of 100 mg/mL before biological assays. Upon resuspension, peptide fractions were diluted under experimental conditions to minimize potential residual effects of pH or salts.

The extraction and hydrolysis procedures were performed following previously described protocols for *P. tricornutum* peptide hydrolysates [18].

### 4.3. Cell Line and Viral Strain

The human alveolar epithelial cell line A549 (ATCC CCL-185, donated by Dr. Bruno Rivas Santiago (Unidad de Investigación Biomédica de Zacatecas, Instituto Mexicano del Seguro Social, Zacatecas 98000, Mexico)) was used for all in vitro experiments. Cells were cultured in RPMI-1640 medium (Biowest, Nuaillé, France; Cat. No. BIO-P0860-N10L) supplemented with 8% fetal bovine serum (FBS) (Biowest, Nuaillé, France; Cat. No. BIO-S1620-500), 1% penicillin, and 1% streptomycin (Biowest, Nuaillé, France; Cat. No. BIO-L00022-100), and maintained at 37 °C in a humidified atmosphere containing 5% CO_2_.

The SARS-CoV-2 Omicron variant JN.1 (B.1.1.529, donated by the Consorcio Mexicano de Vigilancia Genómica (CoViGen-Mex, by Dr. Moisés León Juárez, Mexico City, Mexico) was used for infection assays. Viral stocks were propagated and titrated in VERO cells (CVCL_0059. Kindly donated by Dr. Rosa María del Ángel (Department of Infectomics and Molecular Pathogenesis, CINVESTAV-Zacatenco, Mexico City, Mexico) before use in experiments. Viral titers were determined through infection assays followed by flow cytometry analysis [40].

### 4.4. Biosafety and Regulatory Compliance

All experimental procedures involving SARS-CoV-2 were conducted at the Unidad de Investigación Biomédica de Zacatecas (UIBZ) of the Instituto Mexicano del Seguro Social (IMSS). Cell culture procedures were performed in biosafety level 2 (BSL-2) laboratories, whereas SARS-CoV-2 infection assays were conducted in biosafety level 3 (BSL-3) facilities. Both facilities are equipped to provide maximum protection for personnel, the environment, and the surrounding community.

The study protocol was reviewed and approved by the *Comité Nacional de Investigación Científica* (CNIC) of the IMSS, which integrates the ethics, research, and biosafety committees (registration number R-2024-785-073). All biosafety procedures were performed in accordance with the WHO *Laboratory Biosafety Manual* (4th edition), the *Biosafety in Microbiological and Biomedical Laboratories* (BMBL, 6th edition), and the Mexican Official Standards NOM-087-ECOL-SSA1-2002 and NOM-012-SSA3-2012.

### 4.5. Cell Viability Assay and CC_50_ Determination

Cell viability was evaluated using an MTT colorimetric assay (Cell Proliferation Kit I MTT, Roche, Basel, Switzerland; Cat. No. 11465007001). A549 cells were seeded in 96-well plates at a density of 1 × 10^4^ cells per well and allowed to reach 70–80% confluence before treatment. Cells were exposed to peptide fractions at concentrations ranging from 50 to 1000 µg/mL for 24 h at 37 °C.

Following incubation, MTT reagent (10 µL) was added, and cells were incubated for 4 h according to the manufacturer’s instructions. Formazan crystals were solubilized (100 μL of the solubilization buffer included in the kit), and absorbance was measured at 620 nm using a microplate spectrophotometer. Cell viability was expressed as a percentage relative to untreated control cells [40].

In parallel, cell viability was independently assessed using a resazurin-based assay. Briefly, resazurin (Invitrogen, Carlsbad, CA, USA; Cat. No. R12204) solution (final concentration, 44 µM) was added to each well, and cells were incubated for 2 h at 37 °C. Fluorescence was measured using excitation/emission wavelengths of 560/590 nm.

Additionally, cellular morphology and monolayer integrity were evaluated by bright-field microscopy following treatment with peptide fractions at different concentrations, as shown in **Supplementary Figure S1**. Images were acquired under identical conditions using an inverted microscope at 10× magnification.

The 50% cytotoxic concentration (CC_50_) values were calculated by nonlinear regression analysis using a sigmoidal dose–response model. When 50% cytotoxicity was not reached within the tested concentration range, CC_50_ values were reported as greater than the highest concentration tested (>1000 µg/mL).

### 4.6. Antiviral Activity Assay and IC_50_ Determination

For antiviral assays, A549 cells were seeded in 24-well plates and infected at 70–80% confluence with SARS-CoV-2 Omicron variant JN.1 at a multiplicity of infection (MOI) of 1. Virus adsorption was carried out for 1 h at 37 °C in Hank’s balanced salt solution (Biowest, Nuaillé, France; Cat. No. BIO-P0451-N1L).

After infection, cells were washed and treated for 24 h at 37 °C with *P. tricornutum* peptide fractions separated by molecular weight (10–30 kDa, 5–10 kDa, 3–5 kDa, and <3 kDa) at increasing concentrations (100, 300, 500, and 1000 µg/mL). Lopinavir (Ambeed, Arlington Heights, IL, USA; Cat. No. A330804) was included as a reference antiviral control [24,25] and was tested at concentrations of 6, 9, 12, and 18 µM (corresponding to 3.77–11.32 µg/mL), selected based on prior cytotoxicity assays to ensure ≥80% cell viability. Infected, untreated cells and uninfected cells (mock-infected) were included as positive and negative controls, respectively.

Cells were subsequently harvested, washed with 1X PBS (Gibco, Grand Island, NY, USA; Cat. No. 70013073), fixed with 4% paraformaldehyde (Sigma-Aldrich, St. Louis, MO, USA; Cat. No. P6148-500G) for 30 min at room temperature, and permeabilized with 0.02% saponin (Sigma-Aldrich, St. Louis, MO, USA; Cat. No. 84510-100G) containing 1% FBS. Intracellular viral infection was detected using a monoclonal anti-SARS-CoV-2 spike protein antibody (GeneTex, Irvine, CA, USA; Cat. No. GTX632604; dilution 1:750), followed by incubation with an Alexa Fluor 647-conjugated secondary antibody (Invitrogen, Carlsbad, CA, USA; Cat. No. A-21235; dilution 1:2000).

Samples were analyzed by flow cytometry using a BD FACSCanto™ II cytometer (BD Biosciences, San Jose, CA, USA). Antiviral activity was expressed as the percentage of spike-positive cells relative to untreated infected controls. Dose–response curves were generated for each peptide fraction, and the 50% inhibitory concentration (IC_50_) values were calculated by nonlinear regression using a sigmoidal dose–response model [40]. When 50% inhibition was not achieved within the evaluated concentrations, IC_50_ values were reported as approximate ranges or limits.

### 4.7. Flow Cytometry and Data Analysis

Flow cytometry data were acquired using BD FACSDiva v9.0. Data were subsequently processed and analyzed using FlowJo v11. Infection rates were expressed as the percentage of spike-positive cells relative to total viable cells, where untreated infected cells were defined as 100% infection [40]. Representative dot plots were selected from independent experiments and are shown in **Supplementary Figure S2**.

### 4.8. Statistical Analysis

All experiments were performed in at least three independent biological replicates, each with technical triplicates. Data are presented as mean ± standard error of the mean (SEM). Nonlinear regression analyses for CC_50_ and IC_50_ calculations were performed using RStudio (version 4.3.1) and GraphPad Prism (version 9.5.1).

For cytotoxicity assays, differences among concentrations were analyzed using one-way ANOVA followed by Tukey’s post hoc test, while comparisons between MTT and resazurin assays were evaluated using two-way ANOVA followed by Šídák’s test.

For antiviral assays, statistical comparisons were performed using one-way ANOVA followed by Tukey’s post hoc test. Statistical significance was established at *p* < 0.05.

## 5. Conclusions

This study demonstrates that peptide fractions derived from the marine microalga *P. tricornutum* exhibit in vitro antiviral activity against SARS-CoV-2 (Omicron JN.1 variant) and a moderate, concentration-dependent impact on cellular metabolic activity, while maintaining overall cell viability within an acceptable range for antiviral evaluation in human A549 epithelial cells. The observed antiviral activity depended on molecular weight and concentration, with the larger fractions (10–30 and 5–10 kDa) standing out for their more consistent and reproducible viral inhibition and a more favorable balance between antiviral efficacy and cytotoxicity.

Comparative analysis with the reference antiviral drug lopinavir further supports the potential of peptide fractions, as they exhibited lower cytotoxicity while retaining antiviral activity under equivalent experimental conditions. The inability to calculate robust CC_50_ values reflects that 50% cytotoxicity was not reached within the tested concentration range, consistent with a broad apparent safety margin under the conditions evaluated.

Taken together, these findings position *P. tricornutum* as a promising marine source of bioactive peptides and reinforce the value of integrated in silico–in vitro approaches for the discovery of novel marine-derived antiviral agents.

## Figures and Tables

**Table 1:** Anti-SARS-CoV-2 activity and selectivity index of peptide fractions.

Compound/Fraction	CC_50_ (µg/mL)	IC_50_ (µg/mL)	SI (CC_50_/IC_50_)
Lopinavir (control)	52 (82.72 µM)	10.9 (17.39 µM)	4.77
30–10 kDa	>1000	100–300	>3.3
5–10 kDa	>1000	100–300	>3.3
3–5 kDa	>1000	<100	>10
<3 kDa	>1000	<100	>10

CC_50_: concentration causing 50% reduction in cell viability determined by MTT/resazurin assays. IC_50_: concentration required to inhibit 50% of SARS-CoV-2 infection. SI: selectivity index (CC_50_/IC_50_). CC_50_ values > 1000 µg/mL indicate that 50% cytotoxicity was not reached at the highest concentration tested.

## Data Availability

The original contributions presented in this study are included in the article/Supplementary Material. Further inquiries can be directed to the corresponding authors.

## Supplementary Materials

The following supporting information can be downloaded at: https://www.mdpi.com/article/10.3390/md24040122/s1, Figure S1: Morphological evaluation of A549 cell monolayers treated with *P. tricornutum* peptide fractions. Representative bright-field micrographs of A549 cells treated with peptide fractions of different molecular weight ranges (10–30 kDa, 5–10 kDa, 3–5 kDa, and <3 kDa) at concentrations of 50, 100, 300, 500, and 1000 µg/mL. Images were acquired after treatment under the same experimental conditions used for cytotoxicity assays. Cell morphology and monolayer integrity were visually assessed across all conditions. Figure S2: Representative flow cytometry dot plots of SARS-CoV-2 infection in A549 cells. Intracellular detection of SARS-CoV-2 Spike protein (Alexa Fluor 647) in mock-infected cells, infected untreated controls, and infected A549 cells treated under post-infection conditions with lopinavir (6, 9, 12, and 18 µM) or peptide fractions of *P. tricornutum* (10–30 kDa, 5–10 kDa, 3–5 kDa, and <3 kDa) at concentrations of 100, 300, and 500 µg/mL. Dot plots represent side scatter area (SSC-A) versus fluorescence intensity of Alexa Fluor 647. All plots were acquired under identical instrument settings and are representative of independent experiments.
