# Supplementary material for: In Vitro Validation of Size-Dependent Antiviral Activity of Phaeodactylum tricornutum-Derived Peptide Fractions Against SARS-CoV-2
Source: Mar Drugs. 2026 Mar 25;24(4):122. doi: 10.3390/md24040122 (PMC13118173; doi:10.3390/md24040122)
Supplement: Supplementary file 1 [file marinedrugs-24-00122-s001.zip › marinedrugs-4148971-supplementary.pdf]

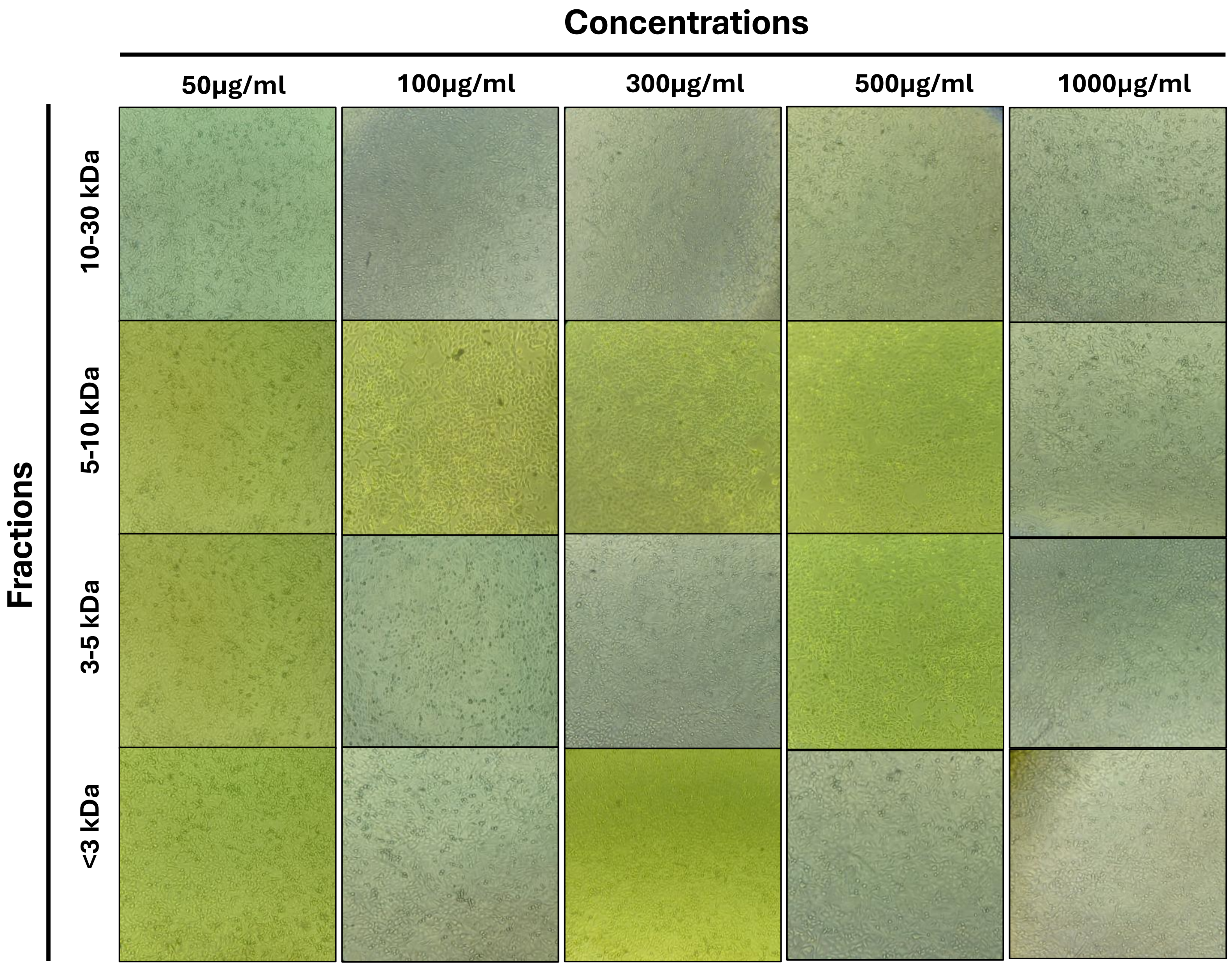

Figure S1. Morphological evaluation of A549 cell monolayers treated with *P. tricornutum* peptide fractions. Representative bright-field micrographs of A549 cells treated with peptide fractions of different molecular weight ranges (10–30 kDa, 5–10 kDa, 3–5 kDa, and <3 kDa) at concentrations of 50, 100, 300, 500, and 1000 μg/mL. Images were acquired after treatment under the same experimental conditions used for cytotoxicity assays. Cell morphology and monolayer integrity were visually assessed across all conditions.

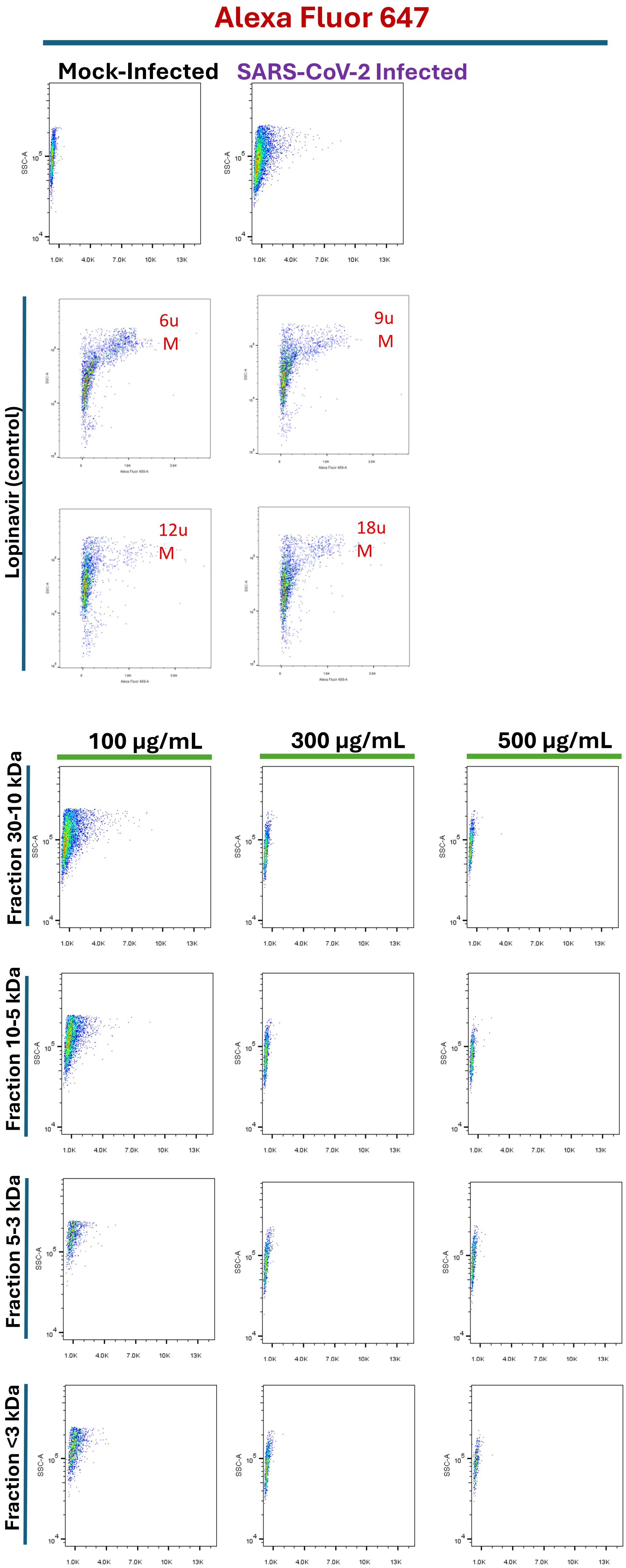

Figure S2. Representative flow cytometry dot plots of SARS-CoV-2 infection in A549 cells. Intracellular detection of SARS-CoV-2 Spike protein (Alexa Fluor 647) in mock-infected cells, infected untreated controls, and infected A549 cells treated under post-infection conditions with lopinavir (6, 9, 12, and 18 µM) or peptide fractions of *P. tricornutum* (10–30 kDa, 5–10 kDa, 3–5 kDa, and <3 kDa) at concentrations of 100, 300, and 500 µg/mL. Dot plots represent side scatter area (SSC-A) versus fluorescence intensity of Alexa Fluor 647. All plots were acquired under identical instrument settings and are representative of independent experiments.
